# Lipid Compositions and Geographical Discrimination of 94 Geographically Authentic Wheat Samples Based on UPLC-MS with Non-Targeted Lipidomic Approach

**DOI:** 10.3390/foods10010010

**Published:** 2020-12-23

**Authors:** Mengchu Jin, Wenhao Zheng, Yaqiong Zhang, Boyan Gao, Liangli (Lucy) Yu

**Affiliations:** 1 Institute of Food and Nutraceutical Science, School of Agriculture and Biology, Shanghai Jiao Tong University, Shanghai 200240, China; jmc0328@sjtu.edu.cn (M.J.); zhengwenhao@sjtu.edu.cn (W.Z.); yqzhang2006@sjtu.edu.cn (Y.Z.); 2 Department of Nutrition and Food Science, University of Maryland, College Park, MD 20742, USA; lyu5@umd.edu

**Keywords:** wheat, lipid profile, LC-MS, non-targeted lipidomic, geographical origins

## Abstract

Wheat is the staple food for the world’s major populations. However, chemical characters of geographically authentic wheat samples, especially for the lipids, have not been deeply studied. The present research aimed to investigate lipid compositions of Chinese wheat samples and clarify the major markers that contribute to the geographical differences. A total of 94 wheat samples from eight main wheat-producing provinces in China were evaluated to differentiate their lipid compositions. Based on the data collected from ultra-high-performance-liquid-chromatography tandem time-of-flight mass spectrometry (UPLC-Q/TOF MS), an optimized non-targeted lipidomic method was utilized for analyses. As the results, 62 lipid compounds, including fatty acids, phospholipids, galactolipids, triglycerides, diglycerides, alkylresorcinol, and ceramide were tentatively identified. Partial least squares discriminant analysis (PLS-DA) demonstrated a more satisfying performance in distinguishing wheat samples from different origins compared with principal component analysis (PCA). Further, the abundances of triglycerides and glycerophospholipids with more unsaturated fatty acids were found greater in wheat samples from northern origins of China, while more glycolipids and unsaturated fatty acids arose in southern original wheat samples. These findings describe the lipid profiles of wheat samples in China and could contribute to the quality and safety control for the wheat flour products.

## 1. Introduction

Wheat is one of the most important cereal crops that has nourished human beings for thousands of years [1]. In China, wheat is recognized as one of three most important grain crops, following rice and maize, with the production at 133 million tons in 2019 [2], and wheat flour is the most widely consumed carbohydrate source in the northern part of China. Hence, the safety and quality of wheat is critical to the national nutrition and health in China. The main high-quality production bases of wheat in China located in Yellow–Huai–Hai River Drainage Basin and North China Plain [3]. Previous publications reported that the botanical origins with diverse geographical features play important roles in determining chemical characteristics of crops and qualities of wheat flour final products [4,5], which also aroused a great deal of attentions toward food traceability [6]. For example, the labels on bread and pasta, especially the ones produced in Italy, must denote the producing regions where the wheat samples were cultivated and milled [7]. In order to clarify the geographic origins of botanical samples, the primary step is to establish the authentic sample information. Hence, it is of crucial importance to discriminate the chemical profiles of geographically authentic samples, so as to better describe the chemical characteristics of the authentic wheat samples.

Lipids are one group of important functional components in wheat grains, with the proportions at 2–2.5% [8]. Lipids have diverse chemical structures and functionalities [9], and play important roles in supplying energy, constructing membrane, and regulating crop metabolisms in wheat grains [10]. On the other hand, the quality of final products of wheat flour is greatly influenced by the lipid types [11]. For example, polar lipids are beneficial to the baking performance of the wheat flour, while some of the non-polar lipids are detrimental [12]. Larger abundance of fatty acids might be a threat to the safety of wheat storage since these fatty acids could easily be oxidated resulting in rancidity [13]. Thus, these minor components were essential to the quality of some loaf products and the wheat safety. The present study focused on wheat lipids instead of other higher-concentrated components such as wheat proteins or starches because this minor fraction has been less investigated to date than the major ones [14,15]. Moreover, from the perspective of omics, some most relevant differences in the upper reaches of omics towards exogenous factors (like growing location, weather and soil composition) might be detected and amplified investigating differences at small-molecule metabolome level [16,17], at which most studies focused on geographical discrimination of crops were investigated [18,19,20]. Previous researches have revealed that wheat lipids could be influenced by geological factors, thus wheat samples harvested from different origins might have specific lipid constitutes, which may affect the processing characteristics [21,22]. However, the specific lipids profile of geographically authentic wheat samples or the discriminated markers among different origins are not clear [7,17]. An effective analytical approach on investigating lipid profiles and exploring characteristics of wheat samples from different origins is constantly being searched for.

Some techniques have been proved effective in differentiating lipid compositions from different origins of samples. Thin layer chromatography (TLC) was previously used to analyze lipids [23], but the disabilities to identify and quantify lipid species had limited the application of TLC in modern food analytical work [8]. Spectroscopic techniques such as nuclear magnetic resonance spectroscopy (NMR) or infrared spectrum (IR) which could provide the chemical structure elucidation were considered as the powerful approach for lipid analyses in a non-destructive manner [5]. While compared to generally used mass spectrometry techniques, spectroscopic approaches revealed the moderate sensitivity [23]. With the advantages of high throughput, sensitivity, and accuracy, chromatography coupled to mass spectrometry has become one of the most suitable techniques applied in food authentication [24]. Gas chromatography coupled to mass spectrometry (GC-MS) was mainly applied in targeted detecting of fatty acids or polar lipid components in wheat [25,26]. Volatile compounds of wheat samples have drawn much attention, and they have been applied in wheat geographical discrimination using the GC-MS approach [27]. Finnie and colleagues quantitatively characterized the polar lipid components of different layers of wheat fractions based on liquid chromatography-mass spectrometry (LC-MS) techniques [28]. Different from the targeted analyses approaches, non-targeted strategies have aroused much interest in food fraud and traceability detections in recent years. Non-targeted strategies focus on the signals formed from entity foodstuff or the whole substances of one molecular species rather than specific chemical components. Certain methods could also effectively lead to the discovery of discriminated markers that contributed most to the differences [29]. The LC-MS approach has satisfactory ability to separate substances, and is capable of providing sufficient high-resolution chemical information, which is pertinent to non-targeted analyses [30]. Previous researches have reported that non-targeted lipidomic LC-MS approaches were applied in distinguishing different varieties of wheat samples [17,31,32] and different layers of wheat fractions [8,9]. Cavanna and colleagues reported through LC-MS approaches, the geographical discrimination of wheat samples from Italy, other European countries and countries out of Europe using chemometrics [7]. However, very few identification results for the markers in wheat samples were reported in previous studies, which resulted in the shortage of discussion in the chemical profiles of wheat samples from different growing regions. Geng and colleagues have identified lipid species in different layers of wheat grain in detail, and the different ionization techniques combined with two ion modes reflected the complex and time-consuming operations during the analyses [9]. All of these published results indicate that appropriate analytical approaches with chemometrics could effectively differentiate the lipid compositions of food samples.

The aim of the present study is to investigate the lipid compositions of wheat samples harvested from eight main wheat-producing origins and elucidate the major markers that contribute to the differences, in an attempt to clarify the lipid profiles of authentic wheat samples in China. Elucidating the chemical characteristics of authentic wheat samples from different origins would be helpful in understanding the potential qualities and applications of wheat samples harvested from different regions of China, thus extending or improving the wheat growing tendency and purpose in China. Further, after creating appropriate mathematic models, lipid profiles of authentic wheats could also be used as the indicators to monitor potential illegal adulteration in wheat or wheat products. All of these above made it meaningful and urgent to focus on the lipid compositions of authentic wheat samples in China.

## 2. Materials and Methods

### 2.1. Materials and Chemical Reagents

In total, 94 wheat samples from eight provinces in China, including Anhui, Fujian, Guizhou, Guangdong, Hubei, Henan, Gansu, and Shaanxi were gifted by local breeding institutes in 2018 and stored at −4 °C before analyses (Table S1).

Methanol, acetonitrile, isopropanol, and formic acid were in LC-MS grade and purchased from Merck KGaA (Darmstadt, Germany). HPLC grade dichloromethane used for extraction was purchased form Sigma-Aldrich (St. Louis, MO, USA). Water was purified by Milli-Q 10 ultrapure water system (Millipore Laboratory, Bedford, MA, USA).

### 2.2. Extraction Methods

Wheat samples were milled with a lab flour grinder (IKA A11 laboratory grinder (IKA, Staufen, Baden-Württemberg, Germany)). A total of 50 mg wheat flour was accurately weighted and mixed with 1.5 mL extraction solution (methanol/dichloromethane (7/3, *v/v*)) in a 2 mL polypropylene centrifugation tube [31]. After vortexed for 60 s, mixtures were extracted in the ultrasonic bath (Hechuang Ultrasonic, Shanghai, China) with the power consumption for 400 W at ambient temperature for 30 min. Extractions were centrifuged (13,000× *g*) at 4 °C for 10 min, and the supernatants were fileted through a 0.22 µm syringe filter. A total of 2 g of each sample was mixed to obtain quality control (QC) sample, and the milling and extraction methods for QC samples were the same as above. Every extraction for QC was prepared along with the same batch of other sample extractions for further data alignment. Each sample was extracted in triplicate.

### 2.3. UPLC Q/TOF-MS Conditions

Waters ACQUITY ultraperformance liquid chromatography combined with a Xevo G2 quadrupole time-of-flight mass spectrometry (UPLC Q/TOF MS) (Waters, Milford, MA, USA) was used for the analyses. The optimized 30-min gradient was carried out with mobile phase A (0.1% formic acid in purified water) and B (0.1% formic acid in isopropanol/acetonitrile (4/6, *v/v*)), and the flow rate was 0.3 mL/min. In total, one aliquot of 2 μL wheat extractions were injected into the Waters Acquity UPLC BEH C18 column (2.1 × 150 mm i.d.; 1.7 μm) with the column temperature at 45 °C. The linear gradient was performed as follows: 0–1 min, 10–50% B; 1–2 min, 50% B; 2–5 min, 50–80% B; 5–8 min, 80–90% B; 8–14 min, 90–91% B; 14–16 min, 91–95% B; 16–18 min, 95–96.5% B; 18–22 min, 96.5–97% B; 22–25 min, 97–100%; 25–28 min, 100% B; 28–28.1 min, 100–10% B; 28.1–30 min, 10% B. The electrospray ionization (ESI) source was set in positive ion mode, with the source temperature at 120 °C and desolvation temperature at 450 °C. The voltages for capillary, sampling cone and extraction cone were 2.5 kV, 40 V, and 4.0 V, respectively. The flow rates of cone gas and desolvation gas were 100 L/h and 800 L/h, respectively. In MS^1^ function, mass range was 100–1200 Da with the collision energy for 6 eV, and the fragment signals under the collision energy ranging from 20–35 eV with the mass of 100–1200 Da in MS^2^.

### 2.4. Data Processing

Chromatograms and MS spectra were acquired by MassLynx 4.1 (Waters, Milford, MA, USA). Identification of chemical compounds in wheat was based on the accurate molecular weight and mass fragment information obtained from MS^1^ and MS^2^ data, theoretical and experimental isotopic patterns, cleavage law of compounds, retention time, as well as the compounds and fragment information reported in previous literatures. Online databases, including SciFinder [33], Pubchem [34], and LIPID MAPS [35] were consulted for features searching.

Before chemometric analyses, Progenesis QI 1.0 (Waters Nonlinear, Milford, MA, USA) was used for data preprocessing, which included peak alignment, experiment design setup, peak picking and compounds identification. One of the QC samples was automatically selected as the most suitable run for peak alignment from all the QCs, then an alpha blend was used to animate between the current and reference runs to make all the runs correctly aligned. After data were grouped by their origins, peak picking was carried out. During the normalization process, one of the runs that is least different from all the other runs in the data set was automatically selected by Progenesis QI to be the normalizing reference. The normalization factor was then calculated by finding the mean of the log abundance ratios of the compounds that fall within the “robust estimated limits”. All compounds were normalized according to their factor based on the reference run. Different adducts of the same compound were then grouped by the deconvolution process. After the compound was identified by QI, the exported data were then delivered to EZinfo (version 3.0, Waters, Milford, MA, USA) for further multivariate data analyses. In the PLS-DA (partial least squares discriminant analysis) loading plot, variables with VIP values > 1.5 (VIP represents variable influence on projection) were selected as significant markers. Box plots that performed by Origin (version 8.5, Originlab, Northampton, MA, USA) were used for capturing abundances of these markers.

## 3. Results and Discussion

### 3.1. Optimization of Analytical Methods

Different extraction methods together with diverse parameters of analytical facilities were explored to obtain better separation effects in the preliminary experiment. Considering the QC sample contained chemical information from all wheat samples and could be recognized as the representative, the optimization of analytical method was carried out by using the QC sample. In order to focus on the lipid components in wheat samples, different extraction reagents, and periods were tested individually. UPLC columns and mobile phases with eluent procedure were also optimized.

Selecting an appropriate extraction solvent plays one of the most important roles in determining the polar-coverage of chemical components from the materials. Therefore, it needs to be carefully optimized [36]. Dichloromethane/methanol (5/5, *v/v*) showed the remarkable capability to extract as many metabolites as possible for wheat matrix compared with n-hexane and acetone/water (5/5, *v/v*). This might be due to the fact that a combination of certain ratios of dichloromethane/methanol covered a large scale of polarity in the metabolites of wheat samples according to the “like dissolves like” rule [37]. In addition, single extraction by dichloromethane/methanol was also compared with the combination of three fractions consequently obtained from one QC sample (Figures S1 and S2). Results showed that the two approaches had no significant difference, which further proved the effectiveness of dichloromethane/methanol. A similar ratio of chloroform/methanol had also been reported suitable for lipidomic analysis of starch-rich samples in some previous publications [31,37]. Besides, different extracting periods (30 min, 60 min, and 120 min) were also compared in their efficiency of extracting non-polar components, and no visual difference was observed among all the three extracting periods, so the final extracting method was determined by using dichloromethane/methanol for 30 min.

Different chromatographic columns including BEH C18, BEH Phenyl, and BEH HILIC were compared with their separation abilities, and the BEH C18 column (2.1 × 150 mm i.d.; 1.7 μm) resulted in the best separation. 0.1% formic acid in water and isopropanol/acetonitrile (4/6, *v/v*) were selected as mobile phase A and B, respectively, based on their stronger elution ability compared with other regents (methanol, acetonitrile). Elution gradient was also optimized to a moderate extent that good separation results can be achieved along with a not too long analytical period in order to give an efficient approach.

### 3.2. Identification of Chemical Compositions in Wheat

**Figure 1:**
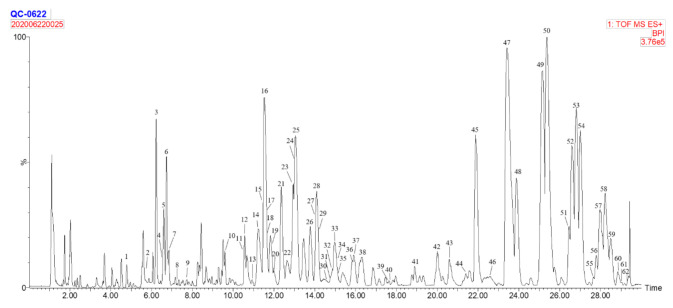
UPLC/Q-TOF-MS BPI chromatogram of lipid profiles in QC wheat sample.

Chemical profiles of wheat were tentatively characterized by UPLC/Q-TOF-MS with the analysis of QC chromatogram. As per the results, a total of 62 components were tentatively identified and numbered in Figure 1, mainly including fatty acids, phospholipids, galactolipids, triglycerides, diglycerides together with a few alkylresorcinol and ceramide (Table 1).

Among all the chemical compounds, polar lipids including phospholipids, galactolipids, few free fatty acids, β-sitosterol, and docosenamide were eluted during the first 16 min, together with alkylresorcinol and ceramide. While in the later 14 min, abundant neutral lipids including diacylglycerols (DGs) and triacylglycerols (TGs) were eluted. Representative lipids of each lipid categories were explained in detail as examples for the identification progresses.

#### 3.2.1. Identification of Fatty Acids

Fatty acids (FAs) were usually found in wheat germ with a content of about 2–5% [38]. In general, FAs were often methylated and analyzed using gas chromatography (GC) [39]. Recently, liquid chromatography combined with high-resolution accurate-mass multistage mass spectrometry (LC-HRAM-MS) have given detailed LC behavior and MS^n^ fragments of FAs in wheat [9,40]. In the present study, the abundance of ions at 279.2325, 263.2378, and 265.2527 m/z in MS^1^ function were much higher than that of MS^2^ and the adduction forms were [M+H]^+^ or [M+H-H_2_O]^+^; thus, three FAs (linolenic acid (C18:3), linoleic acid (C18:2) and oleic acid (C18:1)) were ambiguously identified. Dehydration of the three FAs was observed in the current ESI positive mode of MS fragmentation pathways and the fragments given here (Table 1) were referred to others [9].

#### 3.2.2. Identification of Glycerophospholipids

**Figure 2:**
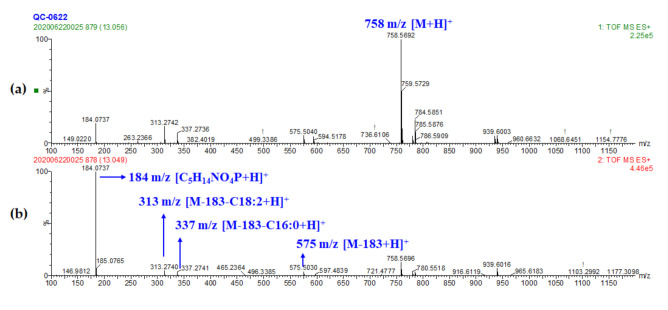
MS spectra of PC (16:0/18:2). (peak 25) (**a**) MS^1^ spectrum and (**b**) MS^2^ spectrum.

Glycerophospholipids were one type of lipids with a glycerol backbone and bound with fatty acids located in *sn*-1 and/or *sn*-2 positions (*sn* represented for stereospecific numbering), and the types of glycerophospholipid were differed by districting polar phosphor groups in the *sn*-3 position [41]. In ESI positive mode, [M+H]^+^ and [M+Na]^+^ were the main molecular ionization mode for phospholipids [41], which were also approved in the present study. A total of eight phosphatidylcholines (PC), two phosphatidylethanolamines (PE), five lyso-phosphatidylcholines (Lyso-PC), and one lyso-phosphatidylglycerol (Lyso-PG) were identified from wheat samples. For PC, the loss of choline moiety resulted in the formation of diagnostic ion [C_5_H_14_NO_4_P+H]^+^ (m/z 184.0739). The types and binding positions of fatty acids could be determined by the diglyceride fragments that formed after losing the polar phosphor group from the PC backbone or fatty acid fragments in the MS^2^ spectrum. For example, peak 25 showed the molecular weight of [M+H]^+^ at m/z 758.5692, so the molecular formula of the certain compound could be calculated as C_42_H_81_NO_8_P within the mass error of 0.67 ppm. Product ion at m/z 184.0737 showed higher abundance in the MS^2^ spectrum referred to the class of phospholipids-phosphatidylcholine (for the number of nitrogen atoms in the molecular formula did not match the composition of sphingomyelin (SM), PC was confirmed). Furthermore, [M-C_5_H_14_NO_4_P+H]^+^, [M-C_5_H_14_NO_4_P-C_18_H_30_O_1_+H]^+^ and [M-C_5_H_14_NO_4_P-C_16_H_30_O_1_+H]^+^ at m/z 575.5029, 313.2741, and 337.2740, respectively, with a slight abundance referred to the identification of palmitic and linoleic acyls (Figure 2). Due to the fatty acids substituent in *sn*-2 position was easier to be eliminated from PC, PG, and PE [41], the distribution of fatty acids can be clarified as PC (16:0/18:2) for the ions at m/z 313.2741 had a higher abundance than 337.2740 and that was reported previously [9,31]. Similarly, polar fragment losses of PG and PE formed [M-172+H]^+^ and [M-141+H]^+^ was observed in peak 5 (Lyso PG (16:0)) and peak 20 (PE (18:2/18:2)) [9,41]. All the other phospholipids were tentatively identified similarly.

#### 3.2.3. Identification of Galactolipids

**Figure 3:**
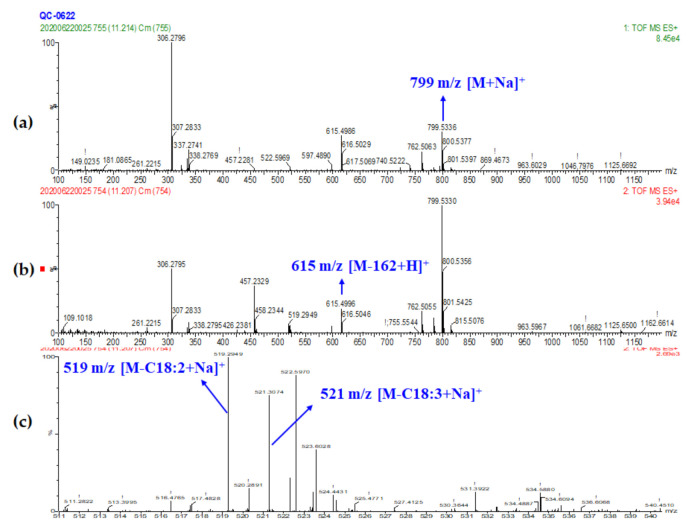
MS spectra of MGDG (18:3/18:2). (peak 14) (**a**) MS^1^ spectrum, (**b**) MS^2^ spectrum and (**c**) Amplificatory MS^2^ spectrum.

Galactolipids were another type of polar lipids as well as a group of bound lipids with mono- or di-galactosyl located in the *sn*-3 position of the glyceride backbone and mainly distributed in wheat endosperm. Monogalactosyl-diacylglycerol (MGDG) and digalactosyl-diacylglycerol (DGDG) were the major galactolipid forms in wheat grain with unsaturated fatty acids that connected to *sn*-1 or/and *sn*-2 position in general [9]. In total, four MGDGs and seven DGDGs were identified in this study with molecular ion forms of [M+Na]^+^ and/or [M+NH_4_]^+^. The main product ions observed in the current positive MS mode were formed by the loss of galactosyl moiety to yield ion [M-162+H]^+^ or/and cleavage of a fatty acid to yield ion [M-RCOOH+Na]^+^ and [M-162-RCOOH+H]^+^. For example, peak 14 with the molecular ion of [M+Na]^+^ at m/z 799.5336 and [M+NH_4_]^+^ at m/z 794.5782 referring to C_45_H_76_O_10_ were both detected in the MS^1^ spectrum (Figure 3). Product ion at 615.4986 represented [M-162+H]^+^, which was formed by the loss of galactosyl and could also be pointed that the fatty acids constitution was C36:5. Furthermore, linoleic acid (C18:2) or linolenic acid (C18:3) losses resulted in fragments of [M-RCOOH+Na]^+^ at m/z 519.2949 and 521.3074, respectively. Due to the fatty acid in the *sn*-2 position of MGDG being more favorable to lose than *sn*-1 [42] and the abundance of 519 m/z was higher than 521 m/z, the distribution of fatty acids for MGDG (18:3/18:2) could be determined.

#### 3.2.4. Identification of Other Minor Polar Lipids

**Figure 4:**
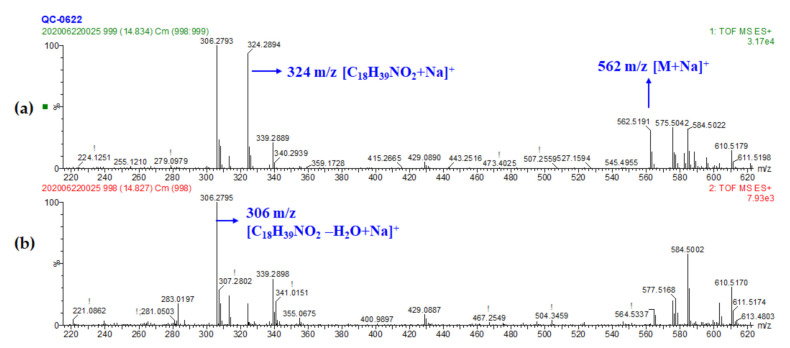
MS spectra of Cer (d18:0/16:0). (peak 30) (**a**) MS^1^ spectrum and (**b**) MS^2^ spectrum.

As the functional constituent of food, sphingolipids have been widely identified in wheat bran [43]. In addition to its structural role, sphingolipids from wheat bran have performed extensive bioactivity [44]. Ceramide (Cer) was the simplest form of sphingolipid with a hydrogen atom substituting for the polar group [45]. Cer (d18:0/16:0) which consisted of a palmitoyl (16:0) and a dihydroxy sphingosine with 18 carbons (d18:0, where d represented two hydroxyls linked to sphingosine) was tentatively identified in the present study with the m/z of 562.5192 for the ion [M+Na]^+^ referring to the molecular formula of C_34_H_69_NO_3_ (peak 30) (Figure 4). Product ions in peak 30 were at m/z of 324.2898 and 306.2794, which corresponded to the fragments of sphingosine (d18:0) according to Cameron [46], so that the constitution of parent ion was clear. However, this was the only ceramide identified in our study. The other variable sphingolipids were not detected probably because of the ionization methods and instrument differences compared with other studies [9,45].

Alkylresorcinols (AR) were amphiphilic phenolic lipids with odd-numbered alkyl chain in the range of C_15_-C_25_ at position 5 of the 1,3-dihydroxybenzene [47]. As the ARs were not easily detected in ESI positive mode compared with ESI negative or APCI positive mode [31], only AR (21:0) (peak 19) were tentatively identified according to the fragments that were reported in previous researches [9].

In addition, other polar lipids including 13-docosenamide (peak 10) and β-sitosterol (peak 13) were also been found and identified according to previous literatures [40].

#### 3.2.5. Identification of Glycerides

**Figure 5:**
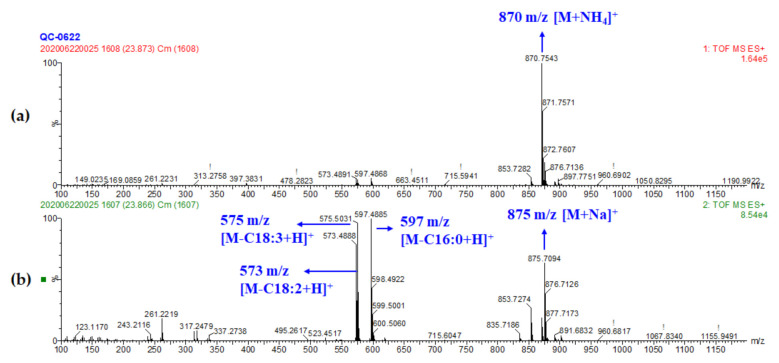
MS spectra of TG (18:3/18:2/16:0). (Peak 48) (**a**) MS^1^ spectrum and (**b**) MS^2^ spectrum.

Triglyceride (TG) and diglyceride (DG) were the main storage lipid distributed in all wheat fractions, especially abundant in the outer layer of wheat kernel [8]. Different from other polar lipids, these neutral lipids eluted finally in the present gradient and showed large structural diversity. The cleavage principle of TGs and DGs were clearly discussed before [48,49]. In this study, a total of 22 TGs and six DGs were tentatively identified. For example, peak 48 revealed the molecular ion of [M+NH_4_]^+^ at m/z 870.7543, referring to its molecular formula C_55_H_96_O_6_. Product ions ([M-RCOOH+H]^+^) showed in MS^2^ were 597.4885, 575.5031, and 573.4888 (relative abundances were 100, 96.47, and 78.59, respectively), corresponding to the loss of palmitic, linolenic, and linoleic acid (Figure 5). Considering fatty acids at *sn*-1 or *sn*-3 with more tendency to be lost, linoleic acid could be identified in the *sn*-2 position [50]. Therefore, peak 48 was identified as TG (18:3/18:2/16:0). Other glycerides were deducted and all summarized in Table 1.

Chemical components identified in the present study revealed large structural diversity comprising of predominant polar and neutral lipids, which also proved the feasibility of our analysis method in non-targeted lipidomic research. However, some lipid groups were not detected, such as oxylipins and γ-oryzanols. In fact, it was too difficult for the single method to detect every compound and unambiguously identify them in a sample [47], the present analysis method may be a simple way to quickly depict the profile of wheat lipids.

### 3.3. Multivariate Modeling for Chemical Characteristics Discrimination of Geographical Authentic Wheat Samples

Chemometrics analyses, especially multivariate models, are usually used in metabolomic researches to explore the discriminations among tremendous amounts of omics data. In the present study, two approaches, principal component analysis (PCA) and partial least squares discriminant analysis (PLS-DA), were used to investigate the chemical differences of geographical authentic wheat samples from eight provinces in China [30].

#### 3.3.1. PCA Analysis

After peak alignment and peak picking achieved by Progenesis QI (Waters, Milford, MA, USA), a total of 3771 variables were detected from 94 wheat samples along with QC samples. Firstly, the raw data consisting of 3771 variables were used for PCA modeling by Pareto scale. Due to the none-clustering information and none-selectivity variable used for the modeling, the unsupervised approach PCA could provide the unbiased general trends of classification characteristics for all the wheat samples (Figure S3). As a result, all the QCs were clustered closely at the center of scores plot, indicating the reliability of data acquisition. In total, 11 total ion chromatography (TIC) of all the overlaid QC runs are listed in Figure S4. Chemical information of wheat from Anhui, Henan, Shaanxi, Fujian, and Guangdong provinces clustered loosely together with QCs which reflected that wheat from these origins contributed more to the commonness from the chemic perspective. Despite the unsatisfactory results obtained from the PCA model, wheat from Gansu province of north China manifested the visible differences against Hubei and Guizhou that came from southern China along the PC1 dimension. In order to achieve better discrimination performance and better find differential substances referring to geographical origins, a supervised cluster approach was further induced.

#### 3.3.2. PLS-DA Analysis

**Figure 6:**
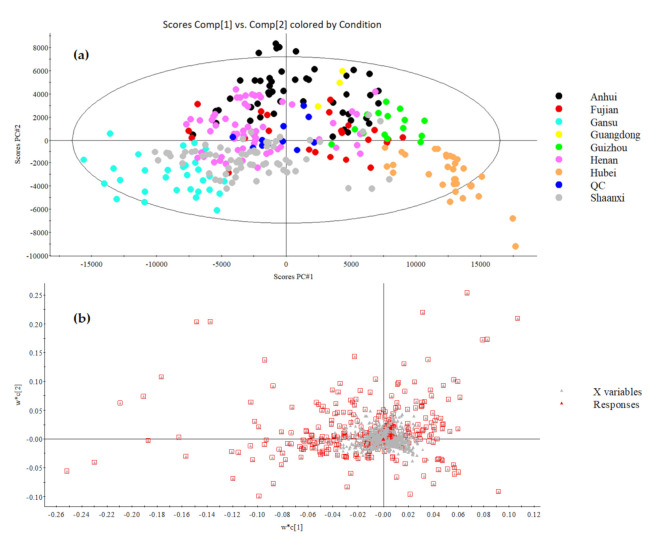
Partial least square discriminant analysis (PLS-DA) (**a**) scores plot and (**b**) loading plot of wheat samples from eight provinces in China. * X variables with the red box in the loading plot were the markers with VIP values > 1.5.

Partial least squares discriminant analysis (PLS-DA) is one of the commonly used supervised models that grouping information was given before modeling. In this study, data with the features *p* < 0.01, coefficient of variance (CV) > 30 and fold change (FC) > 2 was induced for further PLS-DA modeling. Figure 6a visually showed satisfactory discrimination among wheat samples from eight provinces in China based on PLS-DA modeling. In the scores plot of PLS-DA, wheat samples from one province clustered almost tightly. Wheat from Gansu and Hubei displayed the most significant distance along the PC1 dimension, which was consistent with the PCA results. Along the PC2, wheat samples from Anhui and Shaanxi province also revealed a good separation. In addition, the discriminations of sample origins between Gansu and Guizhou, and Gansu and Guangdong province were significant along the diagonal of the first and third quadrants, especially for wheat from Gansu and Guangdong province, the two groups with the longest geographic distance among all wheat samples.

Interestingly, regional factors might play extremely important roles in determining the chemical compositions of wheat samples. The Qinling Mountains–Huaihe River boundary, which traverses from west to east in China, crossing Sichuan, Shaanxi, Hubei, Henan, Anhui, and Jiangsu provinces and connecting the East China Sea, is an essential dividing line separating not only geographic origin and climate but also the social and economic activities such as agricultural production which directly or indirectly affect the foodstuff characteristics [51]. The distinction for chemical profiles of botanical samples from different north-south origins in China was reported previously [5,52,53]. In the present study, most of the wheat samples from north of Qinling Mountains–Huaihe River boundary, including wheat from Shaanxi and Gansu provinces, were clustered on the lower left part of the PLS-DA scores plot, while wheat from southern provinces distributed mainly on the upper right zone, such as wheat from Guizhou and Guangdong provinces (Figure 6a). Meanwhile, wheat samples from central parts of China, including Henan and Anhui, also located at the central of scores plot with slight separation, which further confirmed that growing locations effectively affect the chemical profiles of wheat. However, wheat samples from Fujian province did not show strong clustering into the southern group, which might be for the reason that genotype, cultivation, or other factors that might display an overlap influence on the chemical information discrimination of wheat samples that resulted in the cluster with another irrelevance group set [17]. The clustering characterization depicted the authentic chemical characters of wheat from the specific origin, which gave convincing guidance for wheat origins validation and probably contributed to non-targeted detection of exogenous chemicals. To our best knowledge, this is the first time that a geographical-based distinction of wheat samples across multiple provinces in China has been described based on the perspective of lipid compositions.

Loading plot of PLS-DA reflected the extent that specific variables (chemical substances) contributed to the entirety classification, which meant the variables distributed in the corresponding clustering border with the same location connected to sample in scores plot could be considered as the discriminant markers of this group [54]. In the current study, variables with VIP > 1.5 were selected as the significant markers for the classification and marked with red boxes in the loading plot (Figure 6b), and a total of 317 variables were finally selected as potential markers in differentiating geographical discriminations.

### 3.4. Identified Markers for Wheat Geographical Discrimination

**Figure 7:**
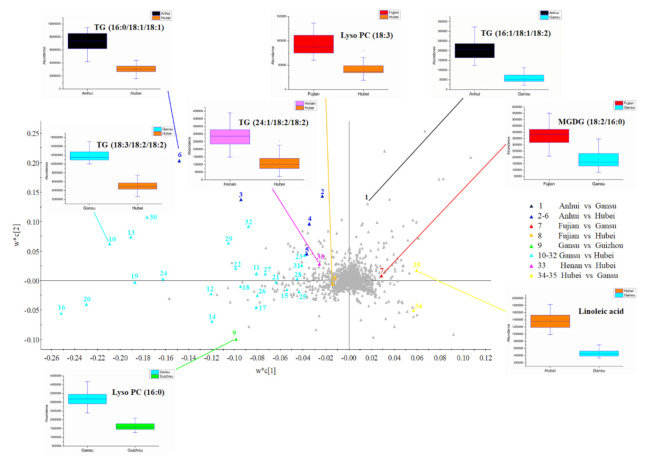
The whole view of the identified discriminated markers * in PLS-DA loading plot. * Markers were these contributed significant difference to the PLS-DA model with VIP > 1.5. Markers with different colors represented the abundance variables between the greatest versus lowest provinces. For example, the abundance of marker No. 1 colored with black was greatest in Anhui province and lowest in Gansu. A total of eight box plots were given for the markers with the maximum VIP value in the corresponding origin group set. Marker numbers and compound details could be found in Table S2.

Among the 317 variables with VIP > 1.5 selected from the PLS-DA loading plot (red boxes in Figure 6b), 35 variables were identified and could be divided into eight groups with statistically meaningful markers. These 35 variables were labeled and identified; the natural abundances of these variables were compared among wheat samples from different provinces (Figure 7, Table S2). Abundances of the markers with the greatest VIP values in each group set were specially performed in Figure 7. For example, the abundances of TG (16:1/18:1/18:2) with the VIP value of 1.87 were greatest in Anhui and lowest in Gansu. Specific abundances of these eight markers in wheat from the provinces with the highest and lowest abundances were listed in Table S3. One thing needed to be mentioned is that although these identified markers showed great differences between the two provinces, they were still significant markers that contributed to differentiate the entire discrimination of wheat samples. From the massive identified marker information, abundances of TGs and glycerophospholipids with more unsaturated fatty acids were found greater in wheat samples from north origins like Gansu and Henan province. Compared with wheat from north origins, southern original wheat samples like Fujian and Hubei contained more glycolipids and unsaturated fatty acids.

Different regions possessed diverse climate situations, including sunshine, precipitation, temperature, and geographical features such as soil condition, altitude, and latitude [5]. These environmental factors led to differences in the metabolic processes of plant, which in turn contributed to the differentiation in metabolites reflected in the clustering model [55]. In China, the important Qinling Mountains–Huaihe River boundary divides the subtropical zone and warm temperate zone, thus the average temperature together with the annual precipitation are higher in the south while lower in the northern part [51].

Among the diverse environmental factors that affected wheat lipid species and contents, temperature, and water stress were reported as essential for the performance of lipidomic [16]. Under higher temperature, plants maintained moderate fluidity and integrity of membranes by re-modelling lipid compositions [21,22]. In the present study, the abundance of PC, PE, MGDG, and DGDG with polyunsaturated acyl chains was lower in the Hubei province, which had a higher average temperature compared with the Gansu province (Table S2). These results were consistent with previous results that decreasing polar lipids like PC, PE, PG, MGDG, DGDG, and SG (sterol glycosides) with two unsaturated fatty acid chains, such as 36:5 or 36:6 when wheat was cultivated under high-temperature stress [22]. TGs with more unsaturated acyl chains were abundant in Gansu, especially TG (18:3/18:2/18:2) performed the highest VIP value of 6.48 in wheat samples from Gansu when compared with that from Hubei province, which was also confirmed as the geographical discrimination marker in the previous publication [32]. Unsaturated fatty acids in wheat lipids were the major *cis* configuration, which added the bends and angulations in fatty acid chains and increased the distances between lipid molecules [22]. Hence, the decrease of unsaturation degree under high-temperature stress was to fasten the membrane structure [21], which was also observed in Henan samples that they contained more TG (24:1/18:2/18:2) compared with Hubei samples. However, linoleic acid and linolenic acid showed higher abundance in Hubei wheat samples compared with Gansu wheat samples, which was contrary to a previous study that the abundance of unsaturated fatty acids was lower under high temperature [21]. That might be the reason why that fatty acids were formed through releasing from membrane lipids in the process of re-modelling lipid compositions to gain more saturated substituents [22].

In addition, greater MGDG (18:2/16:0) abundance was found in Fujian wheat samples while compared with that from Gansu, which might due to the rainfall differences between two provinces according to the previous study that changes of wheat lipid constitutions happened under water shortage treatment [56]. Further, the observed increasing ratio of DGDG and MGDG might be due to the cylindrical shape of DGDG with two galactosyls, which contributed to the formation of a stable bilayer phase while MGDG tended to form an unstable hexagonal phase [10]. These phenomenon might explain the fact that wheat samples from northern provinces in China, which might suffer the water absence induced lower MGDG amount to maintain the stable bilayer membrane structure when compared with wheat samples from southern provinces of China [56].

Diverse lipid constitutes in wheat grains among different regions revealed the adaption that the plant made towards environmental conditions. Thus, the discriminate markers could be seen as the distinct chemical characters of wheat from the specific origin, and could further be used for origin traceability. However, there are still many other factors that might affect the original discriminations and markers of each origin, such as harvest seasons, years, coverage of both producing areas and sample size, and so on. Further, it is difficult to consider all of them in one study [57]. Present results were based on the current sample collection, which collected samples from the main producing areas and controlled the same and main harvest season of wheat in China. The influences of other factors on the original discrimination will be continually investigated in the future.

Although wheat lipids only presented a small amount in the grains, they had contributed significant effects on controlling stability of gas cells in the structures of dough during the fermentation. This effect diversified with different lipid types such as saturated or unsaturated lipids and polar or non-polar lipids [58,59,60]. Understanding the lipid characters of wheat from different origins might be useful for selecting appropriate raw materials for the deep processing of wheat products. Moreover, the presence of free FAs posed a threat to the safety of wheat storage process due to their susceptibility to oxidation and rancidity, which was principally responsible for the decline in quality in food materials [13]. To some extent, the wheat with a higher abundance of FAs in the current research might be paid attention to the safety control during storage process. Additionally, contrary to finding out the markers, a total of 3771 compounds were detected by Progenesis QI software based on the analysis of a total of 94 wheat samples from eight provinces, of which there was 1906 data with features *p* > 0.01, CV < 30, and FC < 2. Therefore, these 1906 compounds could broadly represent the authentic common substance compositions of wheat. In case chemical information of unknown samples differ significantly from the chemical profile we have constructed, there is reason to doubt the correctness of the samples. Conclusively, the lipid profile of authentic wheat samples was captured and the characteristics and commonness of lipids in wheat from different origins were clarified, which enriched the knowledge of wheat chemical constitutes and might provide the chemical information base for wheat quality and safety control.

## 4. Conclusions

To sum up, lipid profiles of authentic wheat samples were determined with UPLC-MS analyses, and characteristics of wheat from eight provinces were investigated using the non-targeted lipidomic approach in the present study. A total of 62 lipid components have been detected and identified from Chinese wheat samples. Polar lipids like phospholipids, galactolipids, and sphingolipids, as well as neutral lipids including triglycerides and diglycerides, took a large proportion and showed diversities among different wheat grains. Supervised multivariate analysis PLS-DA showed remarkable classification of authentic wheat samples harvested from different provinces of China based on lipid compositions, and 35 discriminated markers were identified. The abundance of PC, PE, and some galactolipids with polyunsaturated acyl chains were greater in northern wheat samples, while some southern samples contained more MGDG and probably some fatty acids. The present study established the lipid profiles of authentic wheat samples, proved the differences of lipid compositions in wheat grains from diverse origins, which also revealed the applicability of non-targeted metabolomic approach in establish chemical profiles of botanical crops and markers validation. These findings might also be utilized for breeding and selecting wheat samples with different processing applications.

## Figures and Tables

**Table 1:** Chemical compounds identification of QC * wheat sample.

Peak No.	RT (min)	Exptl. Mass	Calc. Mass	Difference (ppm)	Chemical Formula	Tentative Identification	Adducts #	Fragments
1	4.79	279.2325	279.2324	0.36	C_18_H_30_O_2_	Linolenic acid	M+H	261.2218, 243.2115, 223.1702
2	5.70	518.3240	518.3247	−1.35	C_26_H_48_NO_7_P	Lyso PC (18:3)	M+H, M+Na	184.0731, 261.2234
3	6.21	520.3404	520.3403	0.19	C_26_H_50_NO_7_P	Lyso PC (18:2)	M+H, M+H-H_2_O, M+Na	184.0739, 337.2746, 263.2377
4	6.57	263.2378	263.2375	1.14	C_18_H_32_O_2_	Linoleic acid	M+H-H_2_O	245.2271
5	6.60	485.2899	485.2879	4.12	C_22_H_45_O_9_P	Lyso PG (16:0)	M+H, M+Na	313.2745
6	6.73	496.3403	496.3403	0	C_24_H_50_NO_7_P	Lyso PC (16:0)	M+H, M+Na, M+H-H_2_O	184.0740, 518.3212, 478.3301, 313.2742
7	6.85	522.3562	522.3560	0.38	C_26_H_52_NO_7_P	Lyso PC (18:1)	M+H, M+Na	184.0737, 544.3376, 339.2884
8	7.24	265.2527	265.2531	−1.51	C_18_H_34_O_2_	Oleic acid	M+H-H_2_O	247.2427
9	7.71	524.3718	524.3716	0.38	C_26_H_54_NO_7_P	Lyso PC (18:0)	M+H, M+Na	184.0740, 546.3522, 341.3028
10	9.59	338.3422	338.3423	−0.29	C_22_H_43_ON	13-docosenamide	M+H	321.3154
11	10.53	961.5863	961.5864	−0.10	C_51_H_86_O_15_	DGDG (18:3/18:2)	M+Na, M+NH_4_	615.4992, 335.2579, 337.2722
12	10.56	780.5539	780.5543	−0.51	C_44_H_78_NO_8_P	PC (18:2/18:3)	M+H, M+Na	184.0740, 804.5352, 615.4985, 337,2734, 335.2593
13	10.90	397.3834	397.3834	0	C_29_H_50_O	β-Sitosterol	M+H-H_2_O	261.2611, 243.2099
14 15	11.21 11.50	799.5336 958.6453	799.5336 958.6467	0 −1.46	C_45_H_76_O_10_ C_51_H_88_O_15_	MGDG (18:3/18:2) DGDG (18:1/18:3)	M+Na, M+NH_4_ M+NH_4_. M+Na	615.4988, 519.2949, 521.3074 617.5142, 963.6005, 339.2884, 335.2583, 261.2213
16	11.54	782.5692	782.5700	−1.02	C_44_H_80_NO_8_P	PC (18:2/18:2)	M+H, M+Na	184.0739, 804.5507, 617.5141, 337.2737, 263.2372
17 18	11.72 11.72	756.5547 937.5855	756.5543 937.5864	0.53 −0.96	C_42_H_78_NO_8_P C_49_H_86_O_15_	PC (16:0/18:3) DGDG (16:0/18:3)	M+H, M+Na M+Na, M+NH_4_	184.0737, 778.5366, 573.4870, 313.2751, 335.2583 591.4999, 313.2751, 335.2583
19	11.90	405.3738	405.3733	1.23	C_27_H_48_O_2_	AR (21:0)	M+H	169.0864
20	11.99	740.5226	740.5230	−0.54	C_41_H_74_NO_8_P	PE (18:2/18:2)	M+H	599.5045, 164.0087, 337.2759, 263.2316
21	12.36	801.5483	801.5493	−1.19	C_45_H_78_O_10_	MGDG (18:2/18:2)	M+Na, M+NH_4_	617.5139, 337.2738, 263.2366
22	12.88	965.6168	965.6177	−0.93	C_51_H_90_O_15_	DGDG (18:2/18:1)	M+Na, M+NH_4_	619.5295, 337.2737, 339.2901
23	12.94	784.5849	784.5856	−0.87	C_44_H_82_NO_8_P	PC (18:2/18:1)	M+H, M+Na	184.0738, 601.5189. 337.2745, 339.2901
24	13.00	939.6007	939.6021	−1.49	C_49_H_88_O_15_	DGDG (16:0/18:2)	M+Na, M+NH_4_	593.5148, 313.2745, 337.2736, 263.2358
25	13.04	758.5695	758.5700	−0.67	C_42_H_80_NO_8_P	PC (16:0/18:2)	M+H, M+Na	184.0737, 575.5029, 313.2738, 337.2738, 263.2369, 239.2382
26	13.68	716.5225	716.5230	−0.70	C_39_H_74_NO_8_P	PE (16:0/18:3)	M+H, M+Na	575.5034, 164.0086, 313.2756
27	14.02	803.5647	803.5649	−0.25	C_45_H_80_O_15_	MGDG (18:2/18:1)	M+Na, M+NH_4_	601.5180, 337.2742, 339.2883
28	14.09	639.4965	639.4964	0.16	C_39_H_68_O_5_	DG (18:2/18:2)	M+Na, M+H	337.2743, 263.2377
29	14.14	777.5486	777.5493	−0.90	C_43_H_78_O_10_	MGDG (18:2/16:0)	M+Na, M+NH_4_	575.5027, 337.2739, 313.2733
30	14.82	562.5194	562.5175	3.37	C_34_H_69_NO_3_	Cer (d18:0/16:0)	M+Na	324.2898, 306.2793
31	14.86	786.6000	786.6013	−1.59	C_44_H_84_NO_8_P	PC (18:1/18:1)	M+H, M+Na	184.0738, 603.5320, 339.2904
32	14.88	941.6179	941.6177	0.21	C_49_H_90_O_15_	DGDG (18:1/16:0)	M+Na, M+NH_4_	577.5204, 595.5320, 339.2892, 313.2736
33	14.98	760.5846	760.5856	−1.34	C_42_H_82_NO_8_P	PC (16:0/18:1)	M+H, M+Na	184.0738, 313.2736, 339.2881
34	15.08	734.5682	734.5700	−2.45	C_40_H_80_NO_8_P	PC (16:0/16:0)	M+H, M+Na	184.0738, 313.2724
35	15.13	967.6302	967.6334	3.31	C_51_H_92_O_15_	DGDG (18:0/18:2)	M+Na, M+NH_4_	621.5435, 341.3051, 337.2723
36	15.84	601.5196	601.5196	0	C_39_H_70_O_5_	DG (18:1/18:2)	M+H-H_2_O, M+Na	641.5116, 337.2740, 339.2924
37	15.91	575.5035	575.5039	−0.67	C_37_H_68_O_5_	DG (18:2/16:0)	M+H-H_2_O, M+Na	615.4962, 313.2730, 239.2368, 263.2388, 337.2751
38	16.21	641.5121	641.5121	0.07	C_39_H_70_O_5_	DG (18:2/18:1)	M+Na, M+H-H_2_O	339.2895, 337.2743
39	17.57	603.5346	603.5352	−0.99	C_39_H_72_O_5_	DG (18:1/18:1)	M+H-H_2_O, M+Na	339.2897
40	17.64	577.5201	577.5196	0.87	C_37_H_70_O_5_	DG (16:0/18:1)	M+H-H_2_O, M+Na	339.2900, 313.2752
41	18.90	877.7283	877.7285	−0.23	C_57_H_96_O_6_	TG (18:3/18:2/18:2)	M+H	597.4886, 599.5010, 261.2220, 337.2750
42	20.17	853.7276	853.7285	−1.08	C_55_H_96_0_6_	TG (18:2/18:2/16:1)	M+H	599.5036, 573,4883
43	20.59	892.7370	892.7394	−2.74	C_57_H_94_O_6_	TG (18:2/18:3/18:3)	M+NH_4_, M+Na	597.4885, 595.4723, 261.2222, 263.2357
44	21.43	855.7402	855.7442	−4.67	C_55_H_98_O_6_	TG (16:1/18:1/18:2)	M+H	575.5041, 601.5191, 591.4982, 263.2359
45	21.87	894.7540	894.7551	−1.24	C_57_H_96_O_6_	TG (18:3/18:2/18:2)	M+NH_4_, M+Na, M+H	597.4882, 599.5024, 261.2225, 263.2365
46	22.27	868.7374	868.7394	−2.31	C_55_H_94_O_6_	TG (16:0/18:3/18:3)	M+NH_4_	573.4875, 595.4715, 261.2215, 239.2369
47	23.42	896.7692	896.7707	−1.66	C_57_H_98_O_6_	TG (18:3/18:1/18:2)	M+NH_4_, M+Na, M+H	599.5039,597.4883, 263.2372, 261.2220, 265.2502
48	23.87	870.7543	870.7551	−0.92	C_55_H_96_O_6_	TG (18:3/18:2/16:0)	M+NH_4_, M+H, M+Na	597.4885, 575.5031, 573.4888, 313.2735, 261.2219, 263.2371
49	25.12	898.7851	898.7864	−1.50	C_57_H_100_O_6_	TG (18:1/18:2/18:2)	M+NH_4_, M+Na, M+H	601.5190, 599.5042, 263.2372, 265.2530
50	25.37	872.7695	872.7707	−1.41	C_55_H_98_O_6_	TG (16:0/18:2/18:2)	M+NH_4_, M+Na, M+H	575.5036, 599.5036, 263.2372, 239.2371
51	26.46	926.8163	926.8177	−1.55	C_59_H_104_O_6_	TG (20:1/18:2/18:2)	M+NH_4_, M+Na	629.5507, 599.5030, 263.2381, 293.2830
52	26.59	900.8008	900.8020	−1.33	C_57_H_102_O_6_	TG (18:2/18:1/18:1)	M+NH_4_, M+Na	601.5192, 603.5335, 265.2534, 263.2368
53	26.81	874.7850	874.7864	−1.60	C_55_H_100_O_6_	TG (18:2/18:1/16:0)	M+NH_4_, M+Na	601.5189, 577.5184, 575.5033, 263.2371, 265.2524, 239.2369
54	27.00	848.7700	848.7707	−0.82	C_53_H_98_O_6_	TG (18:2/16:0/16:0)	M+NH_4_, M+Na	575.5038, 551.5039, 263.2372, 239.2381
55	27.66	954.8481	954.8490	−0.90	C_61_H_108_O_6_	TG (18:2/22:1/18:2)	M+NH_4_	657.5815, 599.5043, 263.2386
56	27.79	928.8327	928.8333	−0.67	C_59_H_106_O_6_	TG (18:2/18:1/20:1)	M+NH_4_	601.5201, 631.5652, 629.5511
57	27.97	902.8168	902.8177	−0.95	C_57_H_104_O_6_	TG (20:1/16:1/18:1)	M+NH_4_, M+Na	603.5355, 575.5048, 631.5648
58	28.23	876.8003	876.8020	−1.99	C_55_H_102_O_6_	TG (16:0/18:1/18:1)	M+NH_4_, M+Na	577.5192, 603.5347, 265.2528, 239.2381
59	28.49	850.7857	850.7864	−0.83	C_53_H_100_O_6_	TG (18:1/16:0/16:0)	M+NH_4_, M+Na	577.5188, 551.5038, 239.2375, 265.2530
60	28.84	824.7705	824.7707	−0.23	C_51_H_98_O_6_	TG (16:0/16:0/16:0)	M+NH_4_	551.5038, 239.2386
61	29.04	982.8810	982.8803	0.71	C_63_H_112_O_6_	TG (24:1/18:2/18:2)	M+NH_4_, M+Na	685.6135, 599.5028
62	29.38	930.8482	930.8490	−0.89	C_59_H_108_O_6_	TG (18:1/18:1/20:1)	M+NH_4_	631.5659, 603.5343, 265.2540

* Abbreviations: QC, quality control samples; RT, retention time; Exptl. and Calc. Mass, experimental and calculative m/z of molecular ions; TG, triacylglycerols; DG, diglyceride; AR, alkylresorcinols; PC, phosphatidylcholine; Lyso PC, lysophosphatidylcholines; PE, phosphoethanolamine; Lyso-PG, lyso-phosphatidylglycerol; DGDG, digalactosyldiacylglycerol; MGDG, monogalactosyldiacylglycerol; Cer, ceramide. # Adducts and fragments were given with the most reliable peaks as well as in the order of peak intensity; The most abundant precursor ion signal in MS ^1^ spectrum and its theoretical value were listed as Exptl. and Calc. Mass.

## Data Availability

The data presented in this study are openly available in China Statistical Yearbook-2020 at http://www.stats.gov.cn/tjsj/ndsj/2020/indexch.htm (Reference number 2); SciFinder at https://scifinder.cas.org/scifinder/view/scifinder/scifinderExplore.jsf (Reference number 33); Pubchem at http://pubchem.ncbi.nlm.nih.gov/ (Reference number 34); Home: LIPID MAPS Lipidomics Gateway at http://www.lipidmaps.org/ (Reference number 35).

## Supplementary Materials

The following are available online at https://www.mdpi.com/2304-8158/10/1/10/s1, Figure S1: Base peak intensity chromatogram of QC sample only extracted by dichloromethane/methanol (5/5, *v/v*); Figure S2: Base peak intensity chromatogram of QC sample merged the 3-step extractions (n-hexane—dichloromethane/ methanol (5/5, *v/v*)—acetone/water (5/5, *v/v*)); Figure S3: Principal component analysis (PCA) scores plot of wheat samples from 8 provinces in China, including 3771 variables without any filtrations; Figure S4: Total ion chromatography (TIC) of all the 11 overlaid QC runs; Table S1: Collection information of wheat samples used in this research; Table S2: Identified discriminant markers according to the PLS-DA loadings with VIP > 1.5; Table S3. Normalized abundance data for the 8 markers in wheat from the provinces with the highest and lowest abundances.
