# Supplementary material for: Lipid Compositions and Geographical Discrimination of 94 Geographically Authentic Wheat Samples Based on UPLC-MS with Non-Targeted Lipidomic Approach
Source: Foods. 2020 Dec 23;10(1):10. doi: 10.3390/foods10010010 (PMC7822159; doi:10.3390/foods10010010)
Supplement: Supplementary file 1 [file foods-10-00010-s001.pdf]

Lipid compositions and geographical discrimination of ninety-four geographically authentic wheat samples based on UPLC-MS with non-targeted lipidomic approach

Mengchu Jin [1], Wenhao Zheng [1], Yaqiong Zhang [1], Boyan Gao [1,*] and Liangli (Lucy) Yu [2]

[1] Institute of Food and Nutraceutical Science, School of Agriculture and Biology, Shanghai Jiao Tong University, Shanghai 200240, China; jmc0328@sjtu.edu.cn (M.J.); zhengwenhao@sjtu.edu.cn (W.Z.); yqzhang2006@sjtu.edu.cn (Y.Z.)
[2] Department of Nutrition and Food Science, University of Maryland, College Park, MD 20742, USA; lyu5@umd.edu
* Correspondence: gaoboyan@sjtu.edu.cn; Tel.: +86-021-34204538

Table 1. Collection information of wheat samples used in this research.

Sample No.	Geographical origin (province)
1-16	Anhui
17-24	Fujian
25-29	Guizhou
30	Guangdong
31-41	Hubei
42-61	Henan
62-71	Gansu
72-94	Shaanxi

(All wheat samples were obtained in March 2018, China.)

Table 2. Identified discriminant markers according to the PLS-DA loadings with VIP > 1.5.

Marker No.	m/z	Compound	VIP	Highest abundance	Lowest abundance
1	855.74	TG (16:1/18:1/18:2)*	1.87	Anhui	Gansu
2	824.77	TG (16:0/16:0/16:0)	5.43	Anhui	Hubei
3	850.79	TG (18:1/16:0/16:0)	4.04		
4	930.85	TG (18:1/18:1/20:1)	4.04		
5	967.63	DGDG (18:0/18:2)	1.95		
6	876.80	TG (16:0/18:1/18:1)*	5.81		
7	777.55	MGDG (18:2/16:0)*	2.6	Fujian	Gansu
8	518.32	Lyso PC (18:3)*	1.62	Fujian	Hubei
9	496.34	Lyso PC (16:0)*	4.82	Gansu	Guizhou
10	894.75	TG (18:3/18:2/18:2)*	6.48	Gansu	Hubei
11	641.51	DG (18:2/18:1)	4.04		
12	801.55	MGDG (18:2/18:2)	3.55		
13	870.75	TG (18:3/18:2/16:0)	5.59		
14	780.55	PC (18:2/18:3)	3.8		
15	799.53	MGDG (18:3/18:2)	2.26		
16	782.57	PC (18:2/18:2)	5.88		
17	756.55	PC (16:0/18:3)	2.66		
18	740.52	PE (18:2/18:2)	2.61		
19	784.59	PC (18:2/18:1)	4.33		
20	758.57	PC (16:0/18:2)	5.37		
21	716.52	PC (18:2/13:0)	1.82		
22	786.60	PC (18:1/18:1)	3.89		
23	941.62	DGDG (18:1/16:0)	1.9		
24	760.59	PC (16:0/18:1)	4.32		
25	734.57	PC (16:0/16:0)	1.78		

26	575.50	DG (18:2/16:0)	4.62		
27	892.74	TG (18:2/18:3/18:3)	3.15		
28	868.74	TG (16:0/18:3/18:3)	1.95		
29	926.82	TG (20:1/18:2/18:2)	3.27		
30	848.77	TG (18:2/16:0/16:0)	5.32		
31	954.85	TG (18:2/22:1/18:2)	2.02		
32	928.83	TG (18:2/18:1/20:1)	2.98		
33	982.88	TG (24:1/18:2/18:2)*	1.88	Henan	Hubei
34	279.23	Linolenic acid	2.04	Hubei	Gansu
35	263.24	Linoleic acid*	3.76		

VIP represents variable influence on projection.
Compounds marked with * were those with maximum VIP value of the corresponding province group set.

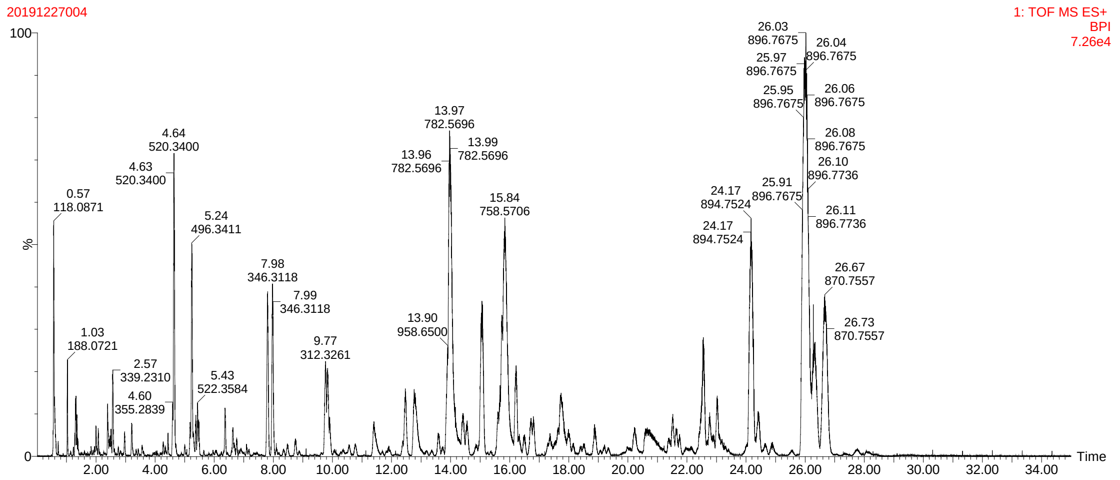

Figure 1. Base peak intensity chromatogram of QC sample only extracted by dichloromethane/methanol (5/5, v/v).

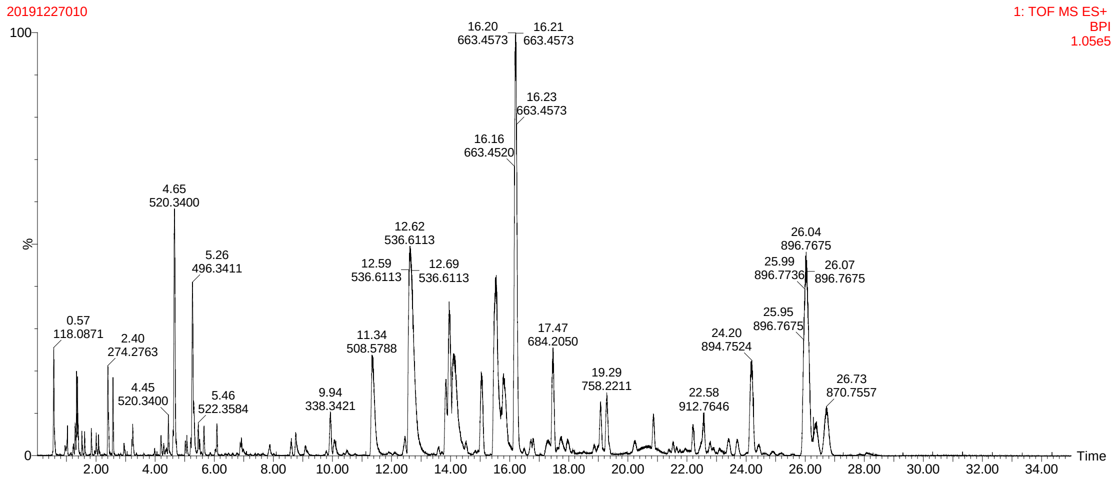

Figure 2. Base peak intensity chromatogram of QC sample merged the 3-step extractions (n-hexane - dichloromethane/methanol (5/5, v/v) - acetone/water (5/5, v/v)).

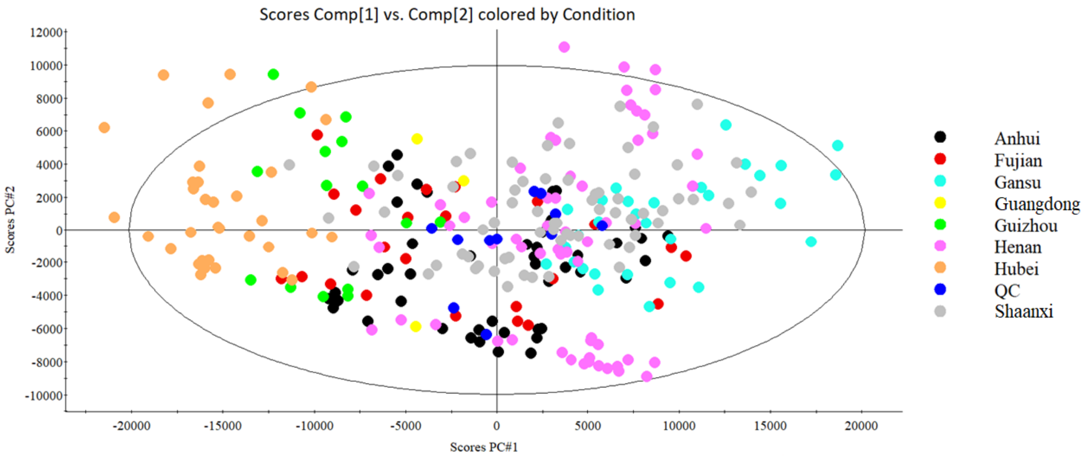

Figure 3. Principal component analysis (PCA) scores plot of wheat samples from 8 provinces in China, including 3771 variables without any filtrations.

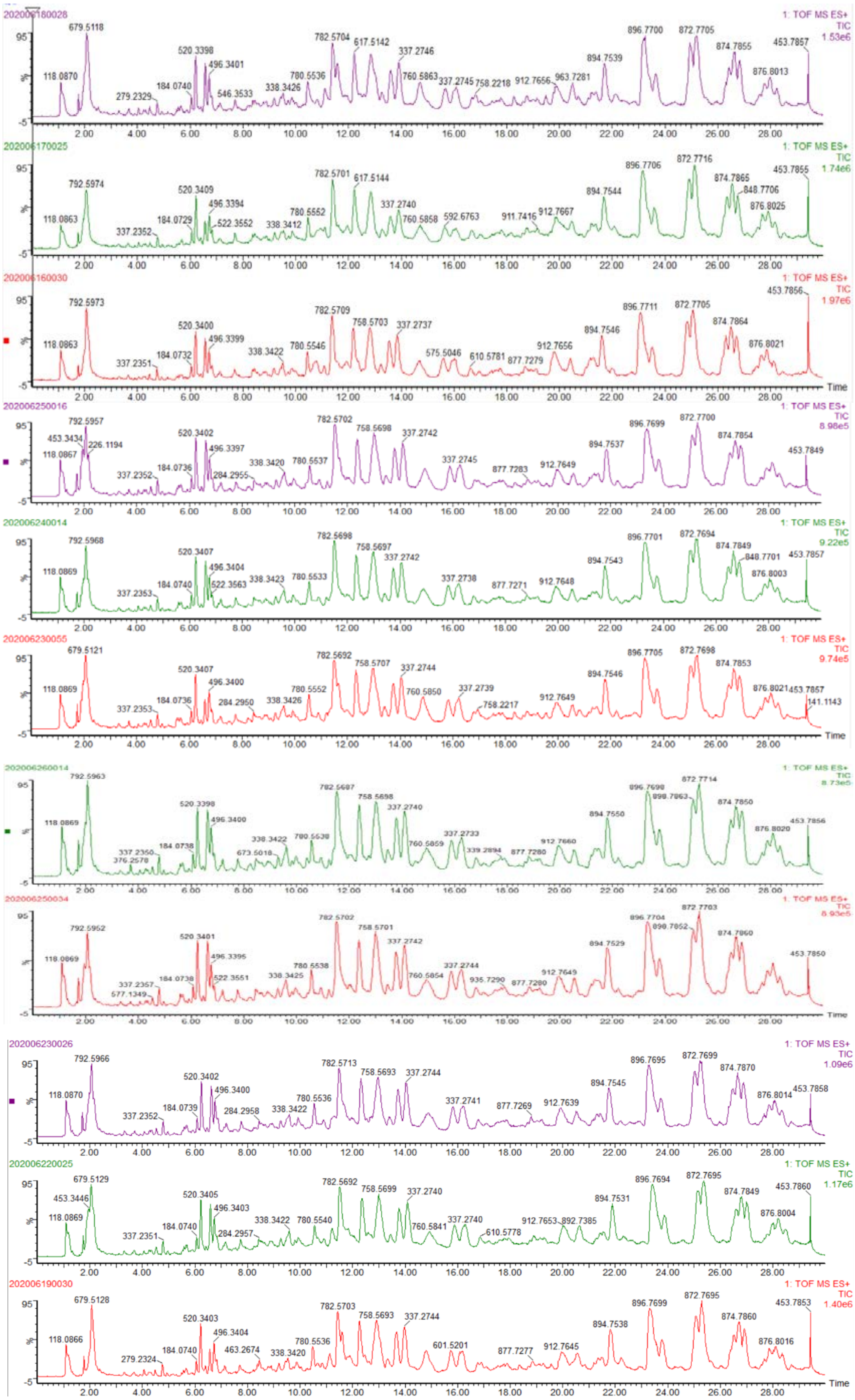

Figure 4. Total ion chromatography (TIC) of all the 11 overlaid QC runs.
